# Deciphering the Diversity of Mental Models in Neurodevelopmental Disorders: Knowledge Graph Representation of Public Data Using Natural Language Processing

**DOI:** 10.2196/39888

**Published:** 2022-08-05

**Authors:** Manpreet Kaur, Jeremy Costello, Elyse Willis, Karen Kelm, Marek Z Reformat, Francois V Bolduc

**Affiliations:** 1 Department of Pediatrics University of Alberta Edmonton, AB Canada; 2 Department of Electrical and Computer Engineering University of Alberta Edmonton, AB Canada; 3 Information Technology Institute University of Social Sciences Łódź Poland; 4 Department of Medical Genetics University of Alberta Edmonton, AB Canada; 5 Women and Children Health Research Institute University of Alberta Edmonton, AB Canada; 6 Neuroscience and Mental Health Research Institute University of Alberta Edmonton, AB Canada

**Keywords:** concept map, neurodevelopmental disorder, knowledge graph, text analysis, semantic relatedness, PubMed, forums, mental model

## Abstract

**Background:**

Understanding how individuals think about a topic, known as the mental model, can significantly improve communication, especially in the medical domain where emotions and implications are high. Neurodevelopmental disorders (NDDs) represent a group of diagnoses, affecting up to 18% of the global population, involving differences in the development of cognitive or social functions. In this study, we focus on 2 NDDs, attention deficit hyperactivity disorder (ADHD) and autism spectrum disorder (ASD), which involve multiple symptoms and interventions requiring interactions between 2 important stakeholders: parents and health professionals. There is a gap in our understanding of differences between mental models for each stakeholder, making communication between stakeholders more difficult than it could be.

**Objective:**

We aim to build knowledge graphs (KGs) from web-based information relevant to each stakeholder as proxies of mental models. These KGs will accelerate the identification of shared and divergent concerns between stakeholders. The developed KGs can help improve knowledge mobilization, communication, and care for individuals with ADHD and ASD.

**Methods:**

We created 2 data sets by collecting the posts from web-based forums and PubMed abstracts related to ADHD and ASD. We utilized the Unified Medical Language System (UMLS) to detect biomedical concepts and applied Positive Pointwise Mutual Information followed by truncated Singular Value Decomposition to obtain corpus-based concept embeddings for each data set. Each data set is represented as a KG using a property graph model. Semantic relatedness between concepts is calculated to rank the relation strength of concepts and stored in the KG as relation weights. UMLS disorder-relevant semantic types are used to provide additional categorical information about each concept’s domain.

**Results:**

The developed KGs contain concepts from both data sets, with node sizes representing the co-occurrence frequency of concepts and edge sizes representing relevance between concepts. ADHD- and ASD-related concepts from different semantic types shows diverse areas of concerns and complex needs of the conditions. KG identifies converging and diverging concepts between health professionals literature (PubMed) and parental concerns (web-based forums), which may correspond to the differences between mental models for each stakeholder.

**Conclusions:**

We show for the first time that generating KGs from web-based data can capture the complex needs of families dealing with ADHD or ASD. Moreover, we showed points of convergence between families and health professionals’ KGs. Natural language processing–based KG provides access to a large sample size, which is often a limiting factor for traditional in-person mental model mapping. Our work offers a high throughput access to mental model maps, which could be used for further in-person validation, knowledge mobilization projects, and basis for communication about potential blind spots from stakeholders in interactions about NDDs. Future research will be needed to identify how concepts could interact together differently for each stakeholder.

## Introduction

Neurodevelopmental disorders (NDDs) are common and represent a group of diagnoses consisting of differences in the development of cognitive, motor, or social skills [1]. Attention deficit hyperactivity disorder (ADHD) is the most common cause of NDDs and affects the ability of children and adults to focus their attention and regulate their motor activity. Another condition is autism spectrum disorder (ASD), which is associated with differences in social interaction, language, and behavior. The prevalence of NDDs is up to 18% worldwide when considering its most common conditions (ADHD) [2,3], while some conditions like ASD will have prevalence closer to 1% [4]. Individuals with ASD and ADHD frequently experience, in addition to their core disorders symptoms, a variety of associated issues, including sleep difficulties, challenging behaviors, and mental health concerns, with repercussions not only on health but also on education and social needs. This creates a level of complexity for parents and a need for large care teams and challenges in communication for health professionals involved with families with NDDs.

Research in medical complexity has shown how communication and care can be improved by establishing each stakeholder’s representation of a condition known as the mental model. Mental models are dynamic and are constantly evolving sets of beliefs and knowledge, which dictate parents’ and professionals’ decisions and behaviors [5,6]. When collaborating with others, having contradictory mental models can lead to conflicting expectations and impede communication [7,8]. Representing mental models visually as a map increases communication and collaboration in education [9] and health care [10]. Mental models have been mapped using various in-person techniques such as cognitive task analysis and concept mapping [11]. Nonetheless, those require trained professionals and access to stakeholders, thereby limiting their scalability.

Knowledge graphs (KGs), as a graph-based information representation format, have been widely applied in artificial intelligence and structural representation of information [12]. KG represents knowledge in a structured way—concepts are nodes connected to each other with edges denoting relationships similar to concept maps. Web-based information has been increasingly used to identify themes of interest to patients. For instance, analysis of web-based information for individuals with cancer has been used to compare patients’ and family members’ concerns [13], patients’ concern and research questionnaires [14], or clinical trial topics [15]. In addition, natural language processing (NLP) techniques have been used to identify and compare the language used to describe different mental health disorders [16]. The word co-occurrence analysis has been used extensively to extract the meanings from text, including health [17], cancer [18], and COVID-19 information, from Twitter [19]. Semantic relatedness tasks play an important role in many NLP applications such as word sense disambiguation [20,21], aspect-based sentiment analysis [22], query expansion [23], and information retrieval from electronic health records [24]. Our study is the first, to our knowledge, to leverage KG building tools to represent mental models from different stakeholders. Moreover, it remains unclear how medical professional literature addresses the topics of most interest to families. Therefore, we propose an approach for comparing ASD-related or ADHD-related concepts that are important and frequently occurring in family forums and in the PubMed literature related to these conditions. Our proposed approach is different from that in the prior mentioned work as it utilizes the vector space model (VSM)–based semantic relatedness technique to construct the KG representation of ASD-related and ADHD-related unified medical language system (UMLS) concepts.

The developed KGs depict concept maps of information from 2 sources: online communities and PubMed abstracts. They help identify concepts with similar and dissimilar relevancy or priority and their frequency of occurrence for the case of both stakeholders. Such a methodology is essential, as obtaining such information directly from stakeholders requires extensive effort involving recruitment and conducting interviews or distributing surveys (with often limited response rate).

## Methods

### Data Collection

#### PubMed Abstracts

Search queries “neurodevelopmental disorders [MeSH],” “autism,” “autism spectrum disorder [MeSH],” “autistic disorder,” “attention deficit and disruptive behavior disorders [MeSH],” “attention deficit disorder with hyperactivity [MeSH],” and “ADHD” were performed in PubMed using Entrez Programming Utilities application programming interface by the National Center for Biotechnology Information. A unique list of 226,660 article identifiers was created, and abstracts were retrieved by making another PubMed application programming interface call, which returned 118,153 nonempty abstracts.

#### Forum Posts

We manually googled publicly available web-based forums or communities and subreddits around the NDD topics to gather social media data and reviewed their privacy policies and terms of use. We selected 3 sources: healthboards.com [25], psychforums.com [26], and reddit [27], for which ethics approval for data collection and analysis was obtained from the University of Alberta. No HTML element containing identifiable personal information such as username was scraped, and only the one containing post was retrieved and stored locally. We did not contact any users for this research. As these online communities are not exclusively focused on NDD topics, we selected subforums about ASD and ADHD such as autism, Asperger syndrome, ADHD, and attention deficit disorder. We found various subreddits around ASD and ADHD, including askAutism, AutismBlogs, TeenAspies, ADD, adhd_anxiety, ADHD, and ParentingADHD. Python Scrapy framework [28] was used to scrape the posts from healthboards.com and psychforums.com, while Reddit application programming interface wrapper [29] was used to collect data from different subreddits. We only considered the main post of the thread and did not collect the list of replies to the thread. We assumed that the main thread consists of the concern posted by the forum user (which was our primary goal in building the KG). We did not include the replies as they would consist of the mention of the same concepts and would falsely boost the frequency of co-occurrence unless the text analysis pipeline has the ability to understand the complete sentence context such as relation extraction task. We did not filter the posts that were posted by parents only; therefore, these could be from any family member, caregiver, a friend of an individual with ASD or ADHD, or an individual with a condition itself.

### NLP Pipeline

#### Data Preprocessing

**Figure 1:**
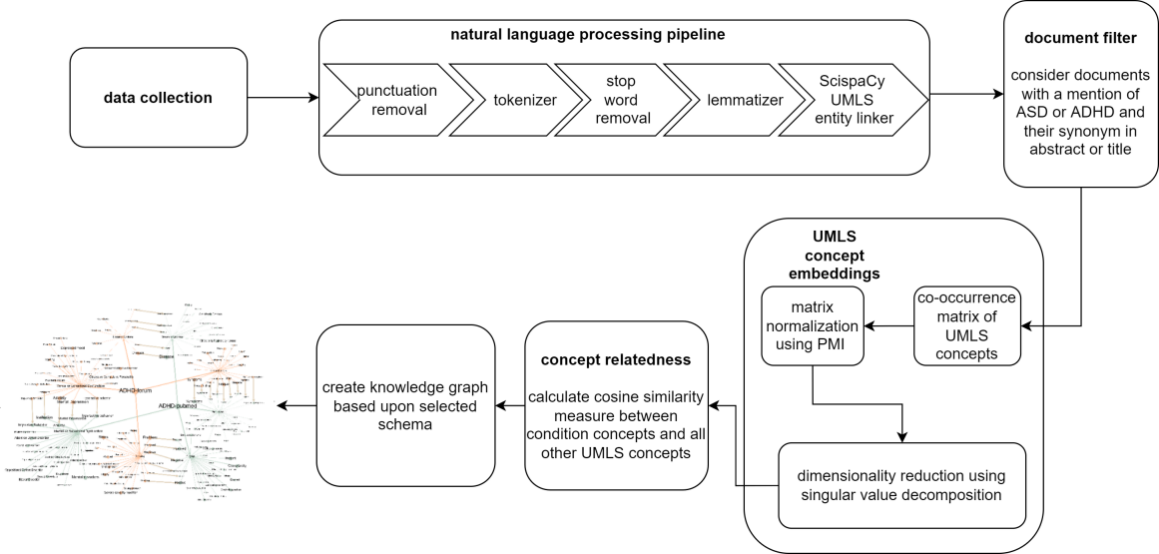
Text processing and knowledge graph generation methodology. Data collection consists of forum scraping using Scrapy, Reddit wrapper application programming interface call, and PubMed application programming interface call. Collected documents are processed through the natural language processing pipeline. The knowledge graph is developed from top 25 concepts related to the condition concepts (autism spectrum disorder or attention deficit hyperactivity disorder) under selected semantic types. ADHD: attention deficit hyperactivity disorder; ASD: autism spectrum disorder; PMI: pointwise mutual information; UMLS: unified medical language system.

All PubMed abstracts and forum posts (henceforth referred to as documents) were preprocessed using the Natural Language Toolkit Python library in order to remove punctuation, tokenize sentences into words, remove stop words, and lemmatize the words [30]. This process is illustrated in Figure 1. Stop words refer to the words that are not informative but occur a number of times such as is, am, are, and have. The default list of stop words provided by the Natural Language Toolkit was used as is.

#### UMLS Entity Linker

The UMLS is a collection of over 100 controlled vocabularies, including but not limited to the International Classification of Diseases-10th classification, medical subject headings, and SNOMED Clinical Terms and contains over 4 million concepts [31]. UMLS facilitates biomedical entity detection by combining synonyms from different source vocabularies into canonical terms called concepts. UMLS also classifies all of its concepts into broader categories called semantic types; for instance, the ASD concept is classified as a mental or a behavioral dysfunction and the training programs concept as an educational activity. Semantic types provide the additional categorical information about the concept and are utilized in this project. An existing open-source Python library scispaCy is used to detect the UMLS concepts from documents [32]. The scispaCy UMLS entity linker provides the score for each detected concept, which ranges from 0 to 1. Low-scored terms would have higher chances of false positives, and we set the probability cutoff of 0.7 to reduce the chances of false positives. Therefore, only the concepts with scores greater than 0.7 along with their semantic type were considered in the final annotation.

In total, 124 UMLS semantic types from PubMed and 122 semantic types from the forum were detected, which could be applicable to all subfields of the medical domain. Peng et al [33] found that the precision of the UMLS entity linker tools could be low if the entities are not specific to ASD, and they used 13 semantic types in their analysis. Our preliminary analysis of all the semantic types was performed by comparing the frequencies of occurrence of each semantic type, which were calculated using all detected concepts from the documents corpus in each source. It showed that the most frequent semantic types such as qualitative concept, functional concept, and idea or concept in the database were not related to ASD and ADHD. Multimedia Appendix 1 shows the top frequent semantic types in each source. Considering the absence of established NDD-related semantic types, we prioritized a set of 26 types by reviewing associated concepts in collaboration with the NDD expert. The selected 26 semantic types are “activity,” “age group,” “behavior,” “congenital abnormality,” “diagnostic procedure,” “daily or recreational activity,” “disease or syndrome,” “educational activity,” “family group,” “finding,” “health care–related organization,” “health care activity,” “individual behavior,” “injury or poisoning,” “mental process,” “mental or behavioral dysfunction,” “occupational activity,” “occupation or discipline,” “organization,” “patient or disabled group,” “professional or occupational group,” “professional society,” “self-help or relief organization,” “social behavior,” “sign or symptom,” and “therapeutic or preventive procedure.” We excluded the frequent semantic types such as qualitative concept, functional concept, and idea or concept from the KG developed for this analysis. However, we are aiming to use those in future works.

If a concept is associated with more than one semantic type, then the scispaCy entity linker returns the list of all semantic types and does not consider the context of the sentences to select the semantic type being discussed. As it returns a list of all semantic types, we considered only the first returned semantic type. Concepts that occur in at least 10 documents in the corpus were considered for further analysis. Thus, we had 4494 unique concepts in PubMed documents and 3627 unique concepts in the forum.

### Document Filter

All documents annotated with UMLS concepts passed through a filter that removed documents without mentioning ASD-related and ADHD-related concepts in the text. In UMLS, ASD, Asperger syndrome, and autistic disorder are different concepts; all the documents that mention any of these in either the abstract or the title are considered under ASD. Further, Asperger syndrome and autistic disorder concepts were replaced with ASD. As a result, we obtained a final data set of 55,461 PubMed abstracts in which 37,728 mentioned ASD, 20,805 mentioned ADHD, and 3072 mentioned both conditions. For the forum, the final data set contained 153,098 posts, in which 72,669 posts were about ASD, 90,372 were about ADHD, and 9943 had statements related to both conditions. Table 1 lists the number of posts collected from 3 web-based forums.

**Table 1:** Number of documents collected from different data sources.

Source	Autism spectrum disorder documents	Attention deficit hyperactivity disorder documents	Both autism spectrum disorder and attention deficit hyperactivity disorder documents
Reddit	66,552	87,022	9302
Psych forums	5029	1966	395
Health boards	1088	1384	246
Total documents from the 3 forums	72,669	90,372	9943
PubMed	37,728	20,805	3072

### UMLS Concept Embeddings

Corpus-based numerical representation of concepts in the VSM represents the meaning of a concept based upon its context. It assumes that concepts that occur together in an environment (either document level, sentence level, or a neighborhood window of a particular size) would be related or similar to each other. The size of the context frames affects the representation of the concepts in the VSM, and many of the word embedding models such as the Skip-gram model and Continuous-bag-of-words model use window-context–based approaches called a local context. Document-level co-occurrence, referred to as a global context, provides more topical information around the concept, as many topic modeling approaches use the global context to detect the latent topics from a document [34]. As we want to detect topically most related concepts to ASD and ADHD, a global context-based co-occurrence matrix of size n × n is created where n refers to the total number of unique UMLS concepts in a source. The co-occurrence matrix is computed separately for PubMed and forum, as contextual information around a concept could be different depending upon the text corpus, which will eventually affect the relatedness scores.

### Positive Pointwise Mutual information

Positive pointwise mutual information (PPMI) followed by truncated singular value decomposition (SVD) is used to embed the concepts, which provide comparative performance to neural network–based embedding models such as Word2Vec [35]. SVD PPMI usually produces consistent/stable results, where stability refers to the change in a word’s neighborhood in the VSM, whereas neural network–based approaches (Word2vec, Glove) could lead to different results in different runs, as the weight of the hidden layers representing the word embeddings differs in multiple runs. SVD-based embeddings are not affected by this problem [36,37]. Pointwise mutual information (PMI) is a probabilistic approach to quantify the likelihood of co-occurrence and tells whether the co-occurrence is informative or by chance. It is defined as follows:

PMI (c_i_, c_j_) = log [p (c_i_, c_j_) / (p (c_i_) × p (c_j_))] **(1)**

where c_i_ = i^th^ concept or the row

c_j_ = j^th^ context concept or the column

p (c_i_) = marginal probability of c_i_

p (c_j_) = marginal probability of c_j_

p (c_i_, c_j_) = marginal probability of c_i_ and c_j_

PMI varies from –1 to 1. If PMI is 0, co-occurrence of 2 concepts does not provide any information and is just by chance. When the joint probability is much higher than marginal probabilities, the co-occurrence is not by chance. If PMI is less than 0, then the independent occurrences of the concepts c_i_ and c_j_ are more informative as compared to co-occurrence. PPMI sets the PMI to 0 if it is less than 0.

PPMI (c_i_, c_j_) = max (PMI (c_i_, c_j_), 0) **(2)**

PPMI provides a square matrix M of size n × n. For the PubMed, n=4494 and for the forum, n=3627, which leads to high dimensionality of the VSM.

### Truncated SVD

SVD is a dimensionality reduction technique used to obtain a low-rank approximation of a dense matrix M. SVD factorizes the matrix M as a product of 3 matrices:

M = USV^T^
**(3)**

where U and V are orthogonal matrices of size n × n and S is a n × n diagonal matrix with diagonal values sorted from high to low. The rank k (k<n) approximation of matrix M can be obtained from equation (3) as follows:

M_k_ = U_k_S_k_V_k_^T^
**(4)**

Where U_k_ is a n × k matrix, S_k_ is a k × k diagonal matrix and V_k_^T^ is a k × n matrix. U_k_S_k_ is the matrix of size n × k, which represents the n concepts in k dimensions. We set k=300 and used Python scikit-learn library to implement truncated SVD and obtain the 300D concept embedding [38]. Different low embedding sizes (usually 300-500) are shown to be used without specific mention of its effect on the final results and 300 dimensions of one of the commonly used sizes [39-41]. PPMI followed by SVD, once applied on forum and PubMed corpus separately, provides 2 VSMs, which represent the concepts depending upon their contextual information in each source.

### Concept Relatedness

Semantic relatedness approaches detect the most related concepts for a given concept based upon the context in which it is used. Semantic similarity and relatedness tasks appear the same, but similarity refers to the concepts that are synonymous and can be used interchangeably, and relatedness refers to concepts that are related because of their usage in the same context. For example, ASD and aggressive behavior are related but not similar. The concept relatedness between 2 concepts c_i_ and c_j_ is measured using cosine similarity as the normalized dot product of the context vectors C_i_ and C_j_:

relatedness_ij_ = cosineSim (C_i_, C_j_) = C_i_ · C_j_ / ║C_i_║║C_j_║

relatedness_ij_ varies from (–1,1), where a value close to 1 means c_i_ and c_j_ are closely related to each other and both vectors have the same orientation in the VSM; a value close to 0 means c_i_ and c_j_ are dissimilar and both vectors are orthogonal in the VSM; and relatedness_ij_ of –1 indicates that c_i_ and c_j_ are in the opposite direction in multidimensional space.

### KG Representation

**Figure 2:**
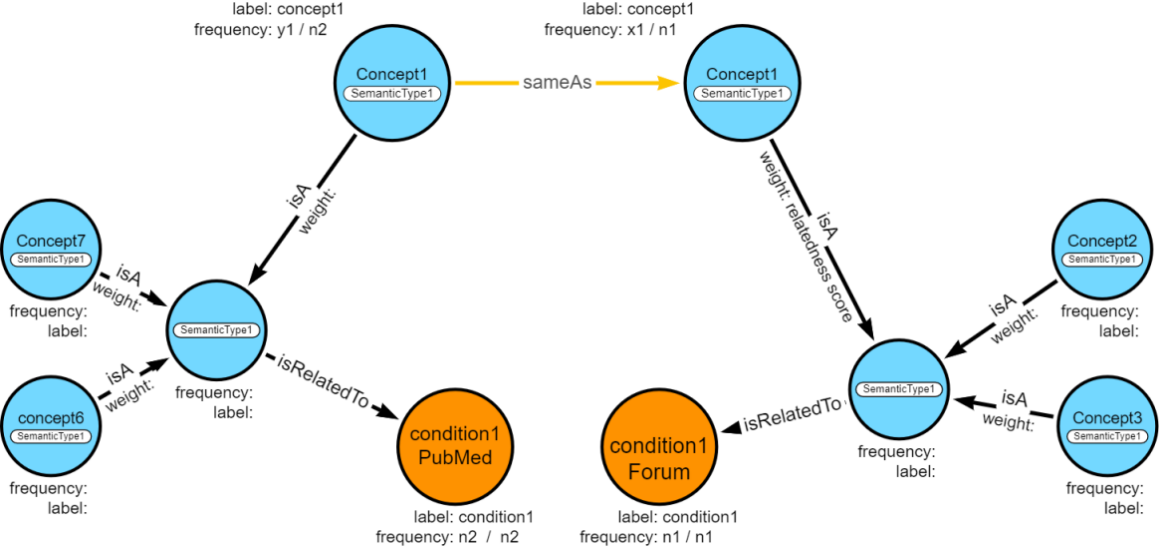
Knowledge graph schema. The co-occurrence frequency of the concept (blue circle) and the condition (orange circle) is stored as the frequency of concept. The relatedness score of the concept to the condition is stored as the weight of isA relationship between the concept and the semantic type (also blue circle). The direction of the sameAs relationship can be either way.

The property graph schema, Figure 2, represents concepts associated with different UMLS semantic terms. There are nodes representing the condition (ASD or ADHD), related UMLS semantic types, and related concepts. Based upon relatedness scores between the condition and the concepts, the top 25 related concepts associated with each UMLS semantic type are used for creating the graph. An edge “isRelatedTo” links a semantic type node to a condition node, and each related concept is connected to its semantic type using the “isA” relationship. A set of property value pairs are stored on nodes as well as edges. All nodes have a label, which refers to the concept name, and the frequency, which is the proportion of documents in which a given concept co-occurred with the condition (ASD or ADHD), in each source data set. The frequency of a semantic type node refers to the average frequency of its top 25 concepts. The weight of the “isA” relationship indicates the relatedness score between the concept and the condition in a source data set, and no weight is assigned to “sameAs” and “isRelatedTo” relations. The Neo4j graph database is used to store the constructed KG [42].

## Results

### Diverse Areas of Concerns Around ADHD and ASD

The developed KG representation of PubMed and forums depict the mental models of both the stakeholders. We found a number of UMLS concepts associated with different semantic types in ADHD-related and ASD-related PubMed and forum data sets. All the detected concepts along with their semantic relatedness score are listed in Multimedia Appendix 2. In order to analyze the different areas of concerns, we assessed health care (PubMed abstracts) and family (forum posts) concepts associated with ADHD by visualizing the KGs from PubMed abstracts and family forums by using the Gephi network visualization tool [43]. In the KG visualizations, the thickness and color darkness of the relationship is proportional to cosine-based relatedness score of the concept to the condition (ADHD or ASD), and the size of the node/label is proportional to the co-occurrence frequency. We detected a few insignificant concepts in some of the semantic type groups. These concepts were then checked against the original text in the PubMed and forum documents, which showed that these concepts were false positives and therefore were removed from all of the analyses. Multimedia Appendix 3 shows the removed concepts along with the frequency of words linked to these concepts. Table 2 summarizes some of the most relevant terms for PubMed and forum documents on ADHD under different UMLS semantic types, which shows the different areas of concern for ADHD.

ADHD KGs generated from PubMed abstracts (see Multimedia Appendix 4) and forums (see Multimedia Appendix 5) show other areas of concerns such as “diagnostic procedure,” “individual behavior,” “health care activity,” and “professional or occupational group.” Similar to ADHD, ASD was found to be linked to diverse concepts in different domains represented by UMLS semantic types as shown in Table 3.

The KG representation of ASD PubMed abstracts (see Multimedia Appendix 6) and forums (see Multimedia Appendix 7) shows concepts under other semantic types, indicating other areas of concerns around ASD.

**Table 2:** Attention deficit hyperactivity disorder–related concepts in PubMed and forums for specific unified medical language system semantic types.

Unified medical language system semantic type	PubMed	Forum
Mental or behavioral dysfunction	Inattention Impulsive behavior Hyperactive behavior Attention deficit disorder Substance abuse problem Conduct disorder	Executive dysfunction Psychiatric problem Anxiety Hyperactive behavior Inattention Mental depression
Age group	Adolescent Adult Young adult	Adult Adolescent Child
Daily or recreational activity	Sports Youth sports Recreational activity	Reading activity Speaking activity Exercise
Educational activity	Psychoeducation Training programs Socialization	Homework Home schooling Training programs
Social behavior	Parenting behavior Social skills Parent-child relationship	Lifestyle Conversation Social behavior

**Table 3:** Autism spectrum disorder–related concepts in PubMed and forums under specific unified medical language system semantic types.

Unified medical language system semantic type	PubMed	Forum
Mental or behavioral dysfunction	Developmental disabilities Social communication disorder Schizophrenia Mental retardation	Bullying Aphasia Social anxiety Stereotypic movement disorder
Age group	Child Adult Infant	Child Adult Adolescent
Social behavior	Communication Social skills Social cognition	Social skills Social situation Eye contact
Mental process	Perception Cognition	Stereotyping Intelligence
Daily or recreational activity	Physical activity Youth sports Speaking and reading activity	Sports Game Speaking and reading activity
Educational activity	Socialization Training programs Computer-assisted instruction Special education Parent training	Socialization Training programs Special education Toilet training Home schooling

### Comparing PubMed and Forum KG

**Figure 3:**
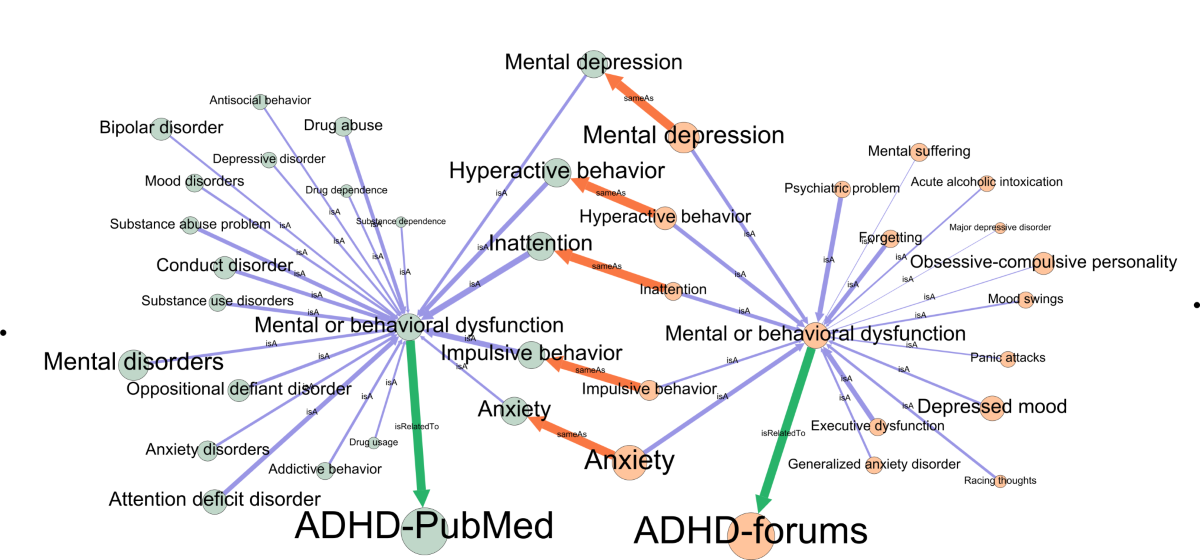
Knowledge graph representation of forums and PubMed around attention deficit hyperactivity disorder for mental or behavioral dysfunction semantic type (green arrow). Concept label font size is proportional to the frequency of the concept in the source. The “sameAs” relationship (orange arrow) connects the overlapping concepts. The thickness of the edge “isA” (purple arrow) refers to the relatedness score of the concept to the condition (attention deficit hyperactivity disorder). ADHD: attention deficit hyperactivity disorder.

KG helps identify concepts of similar and different relevance/priority between families and health professionals. Knowing that shared understanding (shared mental model) has been shown as a key factor in effective collaboration and quality communication in health care [44], we aimed at identifying potential concepts of similar and different relevance between forums and medical literature. For comparing concepts, we considered the top 25 concepts under selected UMLS semantic types, which were the most related to each condition (ASD and ADHD) based upon the relatedness scores, and visualized them using Gephi. As shown in Figure 3, KGs—one for PubMed and one for forum—are connected via the concepts that are of concern for both health professionals and online communities using “*sameA*s” relationship (orange arrow). The direction of this relationship can be either way. For the “*isA”* relationship (purple arrow), its thickness refers to the relatedness score of the concept to the condition (ADHD), which indicates the level of relevance or priority. Different node sizes of concepts connected with “*sameAs”* relationships show differences between the frequency of the concept in respective sources, such as mental depression and anxiety being more commonly discussed in ADHD forums as compared to ADHD PubMed abstracts, while hyperactive behavior, inattention, and impulsive behavior are more discussed in PubMed comparatively.

To summarize the concepts of similar and dissimilar relevance/priority, we compared the relatedness score of all the concepts in forum (FR) and PubMed (PR) and computed the score difference (score difference = FR – PR). The concept is of similar priority if its relatedness score is similar to both stakeholders and score difference of the concept is within µ ± 2σ, where µ is mean and σ is standard deviation of score difference. If score difference > µ + 2σ, then the concept is more relevant for families (forum) and considered as a priority for them because of the substantial score difference. If score difference < µ – 2σ, then the concept is considered as more relevant or as a priority for health professionals (PubMed). Interestingly, as shown in Table 4, we found several concepts of similar and dissimilar relevance to ADHD between PubMed and forum (see Multimedia Appendix 8 for KG visualization). The detailed relevance scores of all these concepts can be found in Tables S1-S3 in Multimedia Appendix 9.

Similarly, comparing the ASD-related concepts in both sources using relatedness score difference and KG representation provided various concepts of similar and dissimilar relevance, as shown in Table 5 (Multimedia Appendix 10 for KG visualization). Detailed relevance scores for all these concepts are listed in Tables S4-S6 in Multimedia Appendix 9.

**Table 4:** Converging and diverging priority level for concepts in attention deficit hyperactivity disorders in PubMed and forum data.

	List of concepts
Concepts with similar relevance for both attention deficit hyperactivity disorder sources	Impulsive behavior Inattention Anxiety Mental depression Hyperactive behavior Sleeplessness Emotional regulation Attention Training programs Socialization
Concepts with high relevance to attention deficit hyperactivity disorder forums	Executive dysfunction Forgetting Racing thoughts Psychiatric problem Nervousness Exhaustion Oversleep Sluggishness Study habits Procrastination
Concepts with high relevance to attention deficit hyperactivity disorder in PubMed	Substance abuse problem Substance dependence Conduct disorder Antisocial behavior Addictive behavior Sleep phase delay Amotivation Anxiety symptoms Oppositional behavior Regulation of behavior

**Table 5:** Converging and diverging priority levels for concepts in autism spectrum disorder in PubMed and forum data sets.

	List of concepts
Concepts with similar relevance for both autism spectrum disorder sources	Social communication disorder Developmental disabilities Aphasia Autistic behavior Intellectual disability Mental disorders Pervasive development disorder Cerebral palsy Seizures Repetitive behavior Social interaction Nonverbal Communication Social behavior Eye contact Social skills Aggressive behavior Self-injurious behavior Stereotyped behavior Behavioral tic
Concepts with high relevance to autism spectrum disorder forums	Bullying Obsessions Social phobia Social anxiety Temper tantrum Mutism Nervousness Social problems Introvert Social life Crowding
Concepts with high relevance to autism spectrum disorder in PubMed	Immune dysregulation Social cognition Behavior adaptive

## Discussion

### Principal Findings

Understanding the needs and concerns of patients and their families is recently being recognized as a key factor for better communication between health professionals and families. This has led to emerging research into the role of mental models in medical practice [45-48] and their mapping [49]. Current approaches include interviews with patients, families, or experts and the identification of main concepts. Crandall et al [6] identified cognitive task analysis as one approach to building mental models. These rich interviews take place over a period of 60-90 minutes with approximately 10 participants. Although the information is rich and in depth, the process is both time-consuming and limited in participant numbers and diversity potentially.

From a theoretical perspective, our work shows how KG building techniques and NLP could help create mental models by using large-scale data sets and avoid bottlenecks such as limited access to experts and privacy/availability for families. Although the NLP methods used are well-established, the use of NLP to generate KGs to derive mental models and to compare them between families and health care professionals’ perspective is completely novel to our knowledge. We show that web-based data from forums capture the diversity of concerns of parents of individuals with 2 important NDDs: ASD and ADHD. Publicly available web-based data could reflect the data obtained from more traditional approaches such as consultations or surveys as published in the literature. We show how using web-based data allows us to identify information about not only diagnostic criteria, medication, symptoms, or comorbidities of a condition but also other areas of concerns such as educational activities, recreational activities, and social issues around a condition, which were usually thought to be accessible mostly by interviews. We also show that the topics are not only related to controversies or unproven therapies, which has often been the rationale for not using web-based information in the medical domain. Similarly, interviews with medical experts are often a bottleneck in understanding concerns in the medical domain.

We also illustrate how web-based data can be used to identify points of convergence in priorities between the different stakeholders involved in complex medical conditions such as ADHD and ASD. Identification of converging points, that is, concepts of similar interest to health professionals and families could help clinicians and extension policy makers to identify “conversation starters” or shared interest. Identifying the diverging concepts or even blind spots for each stakeholder plays an important role for both clinicians and families. For instance, concepts that are highly relevant to families could be used by clinicians to frame continual medication education or training enhancement. For families, they could be the focus of knowledge mobilization, public education campaigns, or further studies aimed at enhancing literacy about their disorder and related conditions.

From a practical point of view, we present a framework that allows us to identify and rank relevant concepts for different sources by using corpus-based embeddings and semantic relatedness approaches as compared to simple co-occurrence frequency to rank related concepts. Developing a KG of the related concepts to represent the mental model visualizations could further assist in comparing converging and diverging concepts between both sources. To our knowledge, as there is no gold standard data set to evaluate the relatedness of concepts in NDDs, our framework proposes to use graph analysis tools such as Gephi to analyze and explore the KG visualizations manually, which could help validate the results by experts. Involving experts (expert in the middle) to review results of NLP approaches facilitates detection of incorrect concepts, which are the result of wrong mapping of abbreviations to concepts. Together, our research provides a proof-of-principle that will generate awareness about KGs as mental model maps and be of use to multidisciplinary researchers in a wide range of medical domains.

### Comparison of KG-Based and Traditional Sources of Information

We compared our findings with previous literature or reports, which are the result of studies using traditional approaches such as interviews or surveys and involving participants (parents or health professionals) from the ASD and ADHD community. For ADHD, for instance, we found that priorities for individuals using the forum (parents, friends, caregivers) were related to prescription of medication and physician types. This reflected what has been discussed in the literature where participating parents were concerned about medication and nonpharmacological interventions (preferred behavior interventions) [50,51]. Another aspect of the topic of health professionals is around the source of information, which was noted previously as a major source of knowledge along with the internet [52]. Focus groups–based study, with caregivers included, showed that the major concern for the parents is about their child becoming a successful adult and improving school behavior [53,54] as well as improving their social situation and emotional state [55], which were identified as a priority before. We found the “behavioral habits” concept with relevancy score of 0.51 as the second most related to ADHD forums in UMLS semantic type “individual behavior.” However, our current approach is based upon UMLS concept recognition and lacks the ability to understand the location as well as age context from the sentences that whether the “behavioral habits” is being discussed for school or home and child or teen. The NLP forum analysis also did not pick on an important trend for parents (and health professionals) to use multimodal interventions [56]. Similarly, our analysis of PubMed papers on ADHD identified topics previously identified by health experts as priorities. We found that the highest ranking topics were discussion of core symptoms of ADHD as well as comorbidities, conduct disorders, and substance use. This mirrors the health experts’ consensus reports highlighting the importance of treatment efficacy for symptoms and raising the point of emotional aspects, academic performance, and work performance [57] as well as comorbidities such as mental illness and substance abuse [58-60]. Overall, we found that the perspectives in family ADHD forums and PubMed papers ranked at similar priority to the core symptoms of ADHD, comorbid conditions such as anxiety and depression, and the educational concerns of training programs and socialization.

With regard to ASD, our other NDD use case in this study, we found that the most overlapping topics had a similar priority level for the different stakeholders reflected by PubMed abstracts and ASD forums. These topics included classification of the condition, symptoms and behaviors that accompany ASD, and topics related to social interaction. Indeed, we found that priorities for people using ASD forums included concerns about social interaction such as social skills, communication, and friendship, as well as daily activities like speaking. This is similar to the findings of a survey distributed by Lai and Weiss [61] investigating service needs for ASD, which found that caregivers prioritized social skills and life-skills programs. Another study also found that the parents’ main concern was social interaction [62], but that study found that the next most prevalent concerns were problem behavior and academics, which we did not see in our analysis of forums. A Serbian study similarly supported communication, social interaction, and daily activities as being caregiver priorities [63]. In addition, our analysis of PubMed abstracts revealed frequent discussion of classification of ASD and its relation to fetal alcohol spectrum and NDDs, concerns about social interaction and communication, and a focus on children with ASD. These priorities are supported by physicians’ approach to ASD, which takes advantage of a diverse team of professionals to focus on improving social interaction and communication [64,65]. This is not to say that parents and research priorities are always aligned as shown in a recent survey in the United Kingdom, illustrating how research tends to be focused on biomedical aspects rather than services and supports [66].

We show that the KG derived from PubMed papers recapitulated the findings of position papers on the topic of ADHD and ASD as mentioned above. However, some of the differences in our findings and the participant-based study results could result from the differences in sample sizes or selection bias (age of caregivers and thus, children could be younger than school or adulthood ages). The collected web-based forum data are considerably larger than the number of participants in interview-based studies and therefore could include points of views not identified before. Alternatively, we could speculate that families may be more inclined to share personal concerns online than in an interview, although we did not find published studies looking into this topic. Further, we have included all the PubMed papers and web-based forums regardless of their publication or posting time (PubMed may include older concepts, which are no longer contemporary concerns), as opposed to the abovementioned expert opinions that were from the last 5 years or less.

### Advantages of Our Approach

Although representing priorities and conceptions of individuals involved in a relation has already been shown to be beneficial to communication and efficacy, using web-based data offers the ability to include a larger number of individuals as shown here from the forum. This would allow for better coverage of the diverse opinion and reflect differences in experience. We also found that forum posts and PubMed papers presented with equivalent density of coverage for all domains examined, suggesting that they present a richness in perspectives and not only trends for instance. Moreover, in the future, our approach could be used to compare concerns of individuals in different countries, in city versus rural settings, or for newcomers to a country, for example. Obtaining the related concepts from the corpus-based VSM and representing those as connected nodes in a property graph model–based KG helps identify convergent and divergent concepts by using different dimensions of interpretability. Node size, which is the frequency of concepts in documents about a condition, tells how widely the concept is discussed in a source. Edge thickness, which is proportional to semantic relatedness score, tells how related a concept is to the condition (ASD or ADHD) depending upon the context in which it is used. This is important as it can help focus attention for knowledge translation and medical education and policy and research development.

### Limitations of Our Approach

Some of the limitations relate to the nature of the data used to construct the graph. Forum posts present some challenges. The forums do not precisely define if the users are parents, caregivers, or potentially family members of individuals with ASD and ADHD. This may influence the type of information requested. In addition, the users are by definition selected on the basis of them using technology to gather information. This could represent a bias based on access to technology, which would be influenced by social determinants of health and therefore could have an incomplete representation of the concerns of parents. In addition, owing to concerns about confidentiality, parents may not share all the concerns they have about their family member with ADHD or ASD. Another important point is that health care is represented by PubMed literature here. Although it is true that PubMed represents a high-quality corpus of medical literature, it may not reflect completely what would be discussed by health care providers, say using web-based forums if they were present. In addition, from a technical standpoint, our proposed semantic relatedness–based KG representation utilizes only the categorical information about the UMLS concepts, which is indicated by the “isA” relationship in KG. However, UMLS provides a semantic network, which shows several meaningful relationships between different semantic types in the form of triples, that is, type1, relation, type2, etc: for instance, (“Mental or Behavioral Dysfunction,” “associated_with,” “Daily or Recreational Activity”) and (“Disease or Syndrome,” “co-occurs_with,” “Mental or behavioral dysfunction”). Utilizing this information could provide more meaningful and direct relations between the concepts of different semantic types. We aim to apply the distantly supervised relation extraction approach on each document corpus, which utilizes the UMLS semantic network to obtain diverse relations between different concepts [67,68]. The output of this approach can also be used as training data for deep learning algorithms to train relation extraction models, which would allow us to create KG by processing text corpus not only for the NDD domain but also for any other condition.

### Conclusion

Our study shows the benefits of using KGs developed based on the results of NLP analysis of a text. The graphs representing the mental models of key concerns from parents of individuals with ASD and ADHD are compared to those built on medical expert knowledge in the same field. The comparison allows identifying points of overlapping and diverging interest. We showed that there are several points of convergence and an extensive list of concerns in both types of stakeholders. This is important, as obtaining such information directly from stakeholders requires extensive effort for recruitment and conducting of interviews or distribution of surveys (with often limited response rate). Furthermore, we found that published reports of polling or interviews with ADHD or ASD families or medical experts identified similar concerns to what we identified through NLP and the comparison of graphs. Future field work would complement our work, which could help understand how different concepts present with complex interactions or how specific populations may differ from one another based on different factors such as social determinants of health.

## Appendix

Multimedia Appendix 1 Bar graph of frequent semantic types in PubMed and forum.

Multimedia Appendix 2 Autism spectrum disorder and attention deficit hyperactivity disorder concept rankings with relatedness scores (multiple sheets).

Multimedia Appendix 3 Mapping of unified medical language system canonical concepts to words in forum and PubMed text.

Multimedia Appendix 4 Knowledge graph of attention deficit hyperactivity disorder PubMed-selected semantic types and concepts (split into subgraphs for clarity).

Multimedia Appendix 5 Knowledge graph of attention deficit hyperactivity disorder forum-selected semantic types and concepts (split into subgraphs for clarity).

Multimedia Appendix 6 Knowledge graph of autism spectrum disorder PubMed-selected semantic types and concepts (split into subgraphs for clarity).

Multimedia Appendix 7 Knowledge graph of autism spectrum disorder forum-selected semantic types and concepts (split into subgraphs for clarity).

Multimedia Appendix 8 Knowledge graph representing similarities and differences between the most relevant attention deficit hyperactivity disorder concepts.

Multimedia Appendix 9 Comparison of concept relatedness scores in forum and PubMed.

Multimedia Appendix 10 Knowledge graph representing similarities and differences between the most relevant autism spectrum disorder concepts.

Multimedia Appendix 11 PubMed abstract data set.

## Abbreviations

ADHD: attention deficit hyperactivity disorder
ASD: autism spectrum disorder
KG: knowledge graph
NDD: neurodevelopmental disorder
NLP: natural language processing
PMI: pointwise mutual information
PPMI: positive pointwise mutual information
SVD: singular value decomposition
UMLS: unified medical language system
VSM: vector space model

## References

[1] Ismail FY, Shapiro BK (2019). What are neurodevelopmental disorders?. Curr Opin Neurol.

[2] Skounti M, Philalithis A, Galanakis E (2007). Variations in prevalence of attention deficit hyperactivity disorder worldwide. Eur J Pediatr.

[3] Polanczyk GV, Salum GA, Sugaya LS, Caye A, Rohde LA (2015). Annual research review: A meta-analysis of the worldwide prevalence of mental disorders in children and adolescents. J Child Psychol Psychiatry.

[4] Chiarotti F, Venerosi A (2020). Epidemiology of Autism Spectrum Disorders: A Review of Worldwide Prevalence Estimates Since 2014. Brain Sci.

[5] Gentner D (2001). Mental Models, Psychology of. International Encyclopedia of the Social & Behavioral Sciences.

[6] Crandall B, Klein G, Hoffman R (2006). Working Minds: A Practitioner's Guide to Cognitive Task Analysis.

[7] Lewis KB, Stacey D, Matlock DD (2014). Making decisions about implantable cardioverter-defibrillators from implantation to end of life: an integrative review of patients' perspectives. Patient.

[8] Holtrop JS, Scherer LD, Matlock DD, Glasgow RE, Green LA (2021). The Importance of Mental Models in Implementation Science. Front Public Health.

[9] Ebener S, Khan A, Shademani R, Compernolle L, Beltran M, Lansang M, Lippman M (2006). Knowledge mapping as a technique to support knowledge translation. Bull World Health Organ.

[10] Zhang C, Zhang J, Long C, Zheng J, Su C, Hu W, Duan Z (2016). Analyses of research on the health of college students based on a perspective of knowledge mapping. Public Health.

[11] Adams S, Nicholas D, Mahant S, Weiser N, Kanani R, Boydell K, Cohen E (2019). Care maps and care plans for children with medical complexity. Child Care Health Dev.

[12] Ji S, Pan S, Cambria E, Marttinen P, Yu PS (2022). A Survey on Knowledge Graphs: Representation, Acquisition, and Applications. IEEE Trans. Neural Netw. Learning Syst.

[13] Andy A, Andy U (2021). Understanding Communication in an Online Cancer Forum: Content Analysis Study. JMIR Cancer.

[14] Ping Qing, Yang Christopher C, Marshall Sarah A, Avis Nancy E, Ip Edward H (2016). Breast Cancer Symptom Clusters Derived from Social Media and Research Study Data Using Improved K-Medoid Clustering. IEEE Trans Comput Soc Syst.

[15] Tapi Nzali Mike Donald, Bringay S, Lavergne C, Mollevi C, Opitz T (2017). What Patients Can Tell Us: Topic Analysis for Social Media on Breast Cancer. JMIR Med Inform.

[16] Gemmell J, Isenegger K, Dong Y, Glaser E, Morain A (2019). Comparing Automatically Extracted Topics from Online Mental Health Disorder Forums.

[17] Jiang L, Yang CC (2013). Using Co-occurrence Analysis to Expand Consumer Health Vocabularies from Social Media Data.

[18] Liu M, Zou X, Chen J, Ma S (2022). Comparative Analysis of Social Support in Online Health Communities Using a Word Co-Occurrence Network Analysis Approach. Entropy (Basel).

[19] Dyer J, Kolic B (2020). Public risk perception and emotion on Twitter during the Covid-19 pandemic. Appl Netw Sci.

[20] Turdakov D, Velikhov P (2008). Semantic Relatedness Metric for Wikipedia Concepts Based on Link Analysis and its Application to Word Sense Disambiguation. http://citeseerx.ist.psu.edu/viewdoc/summary?doi=10.1.1.143.864.

[21] Mao Y, Fung KW (2020). Use of Word and Graph Embedding to Measure Semantic Relatedness Between Unified Medical Language System Concepts. J Am Med Inform Assoc.

[22] Zhou T, Law KM (2022). Semantic Relatedness Enhanced Graph Network for aspect category sentiment analysis. Expert Systems with Applications.

[23] Aggarwal N, Buitelaar P (2012). Query expansion using Wikipedia and Dbpedia. http://ceur-ws.org/Vol-1178/CLEF2012wn-CHiC-AggarwalEt2012.pdf.

[24] Schulz C, Levy-Kramer J, Van AC, Kepes M, Hammerla N (2020). Biomedical concept relatedness: a large EHR-based benchmark.

[25] HealthBoards: Find a Forum.

[26] Psychology and Mental Health Forum.

[27] Reddit.

[28] Scrapy 2.6.

[29] Podolak M PMAW: A Multithread Pushshift.io API Wrapper for Reddit.

[30] Bird S, Klein E, Loper E (2009). Natural Language Processing With Python: Analyzing Text With the Natural Language Toolkit.

[31] Bodenreider O (2004). The Unified Medical Language System (UMLS): integrating biomedical terminology. Nucleic Acids Res.

[32] Neumann M, King D, Beltagy I, Ammar W (2019). ScispaCy: Fast and robust models for biomedical natural language processing.

[33] Peng J, Zhao M, Havrilla J, Liu C, Weng C, Guthrie W, Schultz R, Wang K, Zhou Y (2020). Natural language processing (NLP) tools in extracting biomedical concepts from research articles: a case study on autism spectrum disorder. BMC Med Inform Decis Mak.

[34] Xun G, Li Y, Gao J, Zhang A (2017). Collaboratively improving topic discovery and word embedding by coordinating global and local contexts.

[35] Levy O, Goldberg Y (2014). Neural word embedding as implicit matrix factorization. https://proceedings.neurips.cc/paper/2014/hash/feab05aa91085b7a8012516bc3533958-Abstract.html.

[36] Hellrich J, Hahn U (2017). Exploring Diachronic Lexical Semantics with JeSemE. https://aclanthology.org/P17-4006/.

[37] Chugh M, Whigham P, Dick G (2018). Stability of word embeddings Using Word2Vec.

[38] Pedregosa F, Varoquaux G, Gramfort A, Thirion B, Michel V, Thirion B, Grisel O, Blondel M, Prettenhofer P, Weiss R, Dubourg V, Vanderplas J, Passos A, Cournapeau D, Brucher M, Perrot M, Duchesnay E (2011). Scikit-learn: Machine Learning in Python. Journal of Machine Learning Research.

[39] Hamilton WL, Leskovec J, Jurafsky D (2016). Diachronic Word Embeddings Reveal Statistical Laws of Semantic Change.

[40] Salle A, Villavicencio A, Idiart M (2016). Matrix Factorization Using Window Sampling and Negative Sampling for Improved Word Representations.

[41] Nguyen KA, Schulte IWS, Vu NT (2018). Introducing two Vietnamese Datasets for Evaluating Semantic Models of (dis-)Similarity and Relatedness.

[42] Neo4j Graph Data Platform.

[43] Bastian M, Heymann S, Jacomy M (2009). Gephi: An Open Source Software for Exploring and Manipulating Networks. ICWSM.

[44] Seo S, Kennedy-Metz LR, Zenati MA, Shah JA, Dias RD, Unhelkar VV (2021). Towards an AI Coach to Infer Team Mental Model Alignment in Healthcare. IEEE CogSIMA (2021).

[45] Sturgiss E, Luig T, Campbell-Scherer DL, Lewanczuk R, Green LA (2019). Using Concept Maps to compare obesity knowledge between policy makers and primary care researchers in Canada. BMC Res Notes.

[46] McComb S, Simpson V (2014). The concept of shared mental models in healthcare collaboration. J Adv Nurs.

[47] Mogford RH (1997). Mental Models and Situation Awareness in Air Traffic Control. The International Journal of Aviation Psychology.

[48] Mathieu JE, Heffner TS, Goodwin GF, Salas E, Cannon-Bowers JA (2000). The influence of shared mental models on team process and performance. J Appl Psychol.

[49] Davies M (2010). Concept mapping, mind mapping and argument mapping: what are the differences and do they matter?. High Educ.

[50] Johnston C, Hommersen P, Seipp C (2008). Acceptability of behavioral and pharmacological treatments for attention-deficit/hyperactivity disorder: relations to child and parent characteristics. Behav Ther.

[51] Clarke JN, Lang L (2012). Mothers whose children have ADD/ADHD discuss their children's medication use: an investigation of blogs. Soc Work Health Care.

[52] Bussing R, Zima BT, Mason DM, Meyer JM, White K, Garvan CW (2012). ADHD knowledge, perceptions, and information sources: perspectives from a community sample of adolescents and their parents. J Adolesc Health.

[53] Ross M, Bridges JFP, Ng X, Wagner LD, Frosch E, Reeves G, dosReis S (2015). A best-worst scaling experiment to prioritize caregiver concerns about ADHD medication for children. Psychiatr Serv.

[54] Ross M, Nguyen V, Bridges JFP, Ng X, Reeves G, Frosch E, dosReis S (2018). Caregivers' Priorities and Observed Outcomes of Attention-Deficit Hyperactivity Disorder Medication for Their Children. J Dev Behav Pediatr.

[55] Mühlbacher Axel C, Rudolph I, Lincke H, Nübling Matthias (2009). Preferences for treatment of Attention-Deficit/Hyperactivity Disorder (ADHD): a discrete choice experiment. BMC Health Serv Res.

[56] Lu SV, Leung BMY, Bruton AM, Millington E, Alexander E, Camden K, Hatsu I, Johnstone JM, Arnold LE Parents' priorities and preferences for treatment of children with ADHD: Qualitative inquiry in the MADDY study. Child Care Health Dev.

[57] Ferguson JH (2000). National Institutes of Health Consensus Development Conference Statement: diagnosis and treatment of attention-deficit/hyperactivity disorder (ADHD). J Am Acad Child Adolesc Psychiatry.

[58] Colvin MK, Stern TA (2015). Diagnosis, evaluation, and treatment of attention-deficit/hyperactivity disorder. J Clin Psychiatry.

[59] Jangmo A, Stålhandske Amanda, Chang Z, Chen Q, Almqvist C, Feldman I, Bulik CM, Lichtenstein P, D'Onofrio B, Kuja-Halkola R, Larsson H (2019). Attention-Deficit/Hyperactivity Disorder, School Performance, and Effect of Medication. J Am Acad Child Adolesc Psychiatry.

[60] Lambez B, Harwood-Gross A, Golumbic EZ, Rassovsky Y (2020). Non-pharmacological interventions for cognitive difficulties in ADHD: A systematic review and meta-analysis. J Psychiatr Res.

[61] Lai JKY, Weiss JA (2017). Priority service needs and receipt across the lifespan for individuals with autism spectrum disorder. Autism Res.

[62] Azad G, Mandell DS (2016). Concerns of parents and teachers of children with autism in elementary school. Autism.

[63] Pejovic-Milovancevic M, Stankovic M, Mitkovic-Voncina M, Rudic N, Grujicic R, Herrera AS, Stojanovic A, Nedovic B, Shih A, Mandic-Maravic V, Daniels A (2018). Perceptions on Support, Challenges and Needs among Parents of Children with Autism: the Serbian Experience. Psychiatr Danub.

[64] Lee PF, Thomas RE, Lee PA (2015). Approach to autism spectrum disorder: Using the new DSM-V diagnostic criteria and the CanMEDS-FM framework. Can Fam Physician.

[65] Knott F, Dunlop A, Mackay T (2006). Living with ASD: how do children and their parents assess their difficulties with social interaction and understanding?. Autism.

[66] Pellicano E, Dinsmore A, Charman T (2014). What should autism research focus upon? Community views and priorities from the United Kingdom. Autism.

[67] Zhu T, Qin Y, Xiang Y, Hu B, Chen Q, Peng W (2021). Distantly supervised biomedical relation extraction using piecewise attentive convolutional neural network and reinforcement learning. J Am Med Inform Assoc.

[68] Xing R, Luo J, Song T (2020). BioRel: towards large-scale biomedical relation extraction. BMC Bioinformatics.

[69] National library of medicine terms and conditions. National Library of Medicine.

