# Supplementary figures and images for: Deciphering the Diversity of Mental Models in Neurodevelopmental Disorders: Knowledge Graph Representation of Public Data Using Natural Language Processing
Source: J Med Internet Res. 2022 Aug 5;24(8):e39888. doi: 10.2196/39888 (PMC9391978; doi:10.2196/39888)

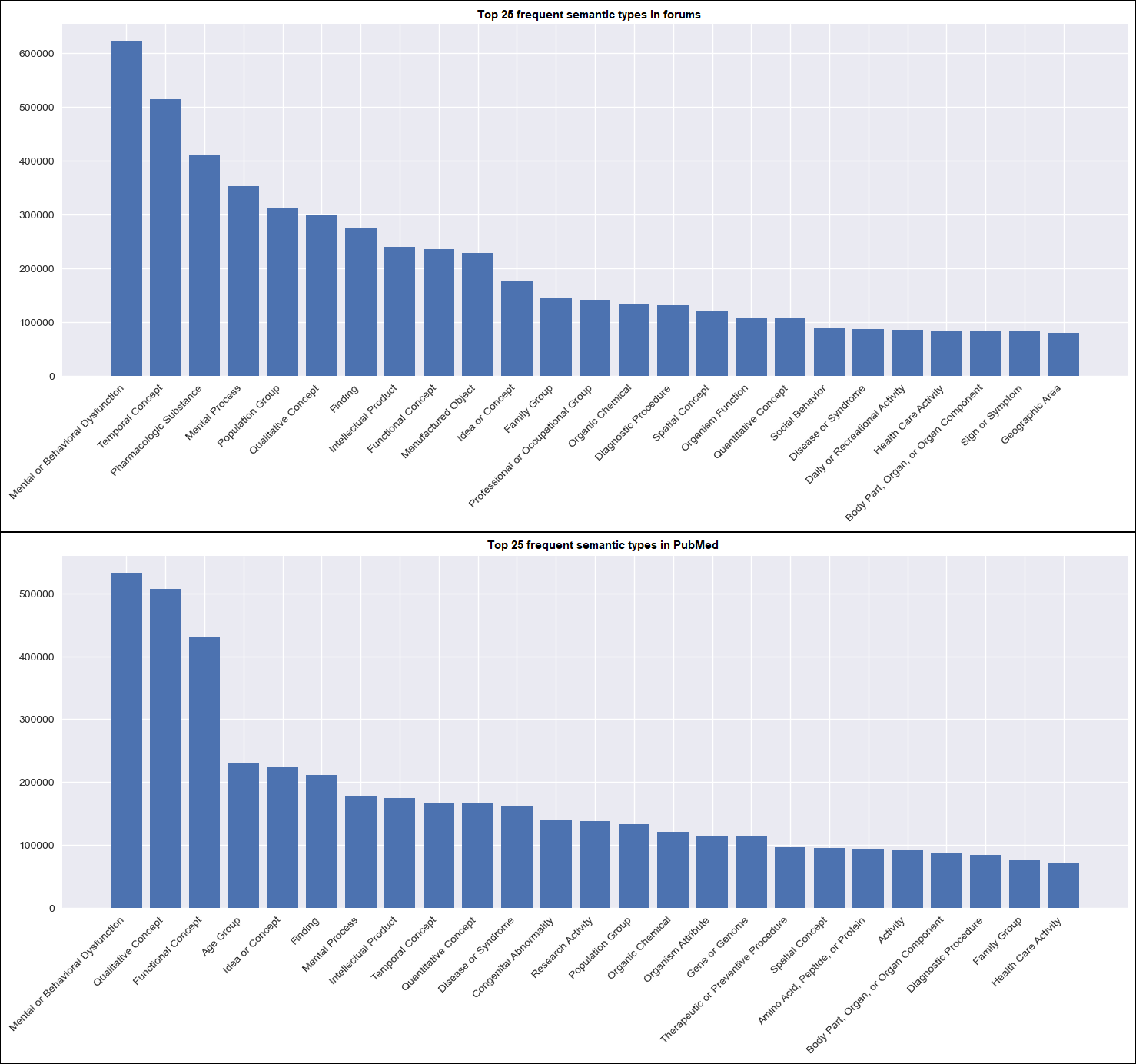

Supplement: Multimedia Appendix 1 [file jmir_v24i8e39888_app1.png]

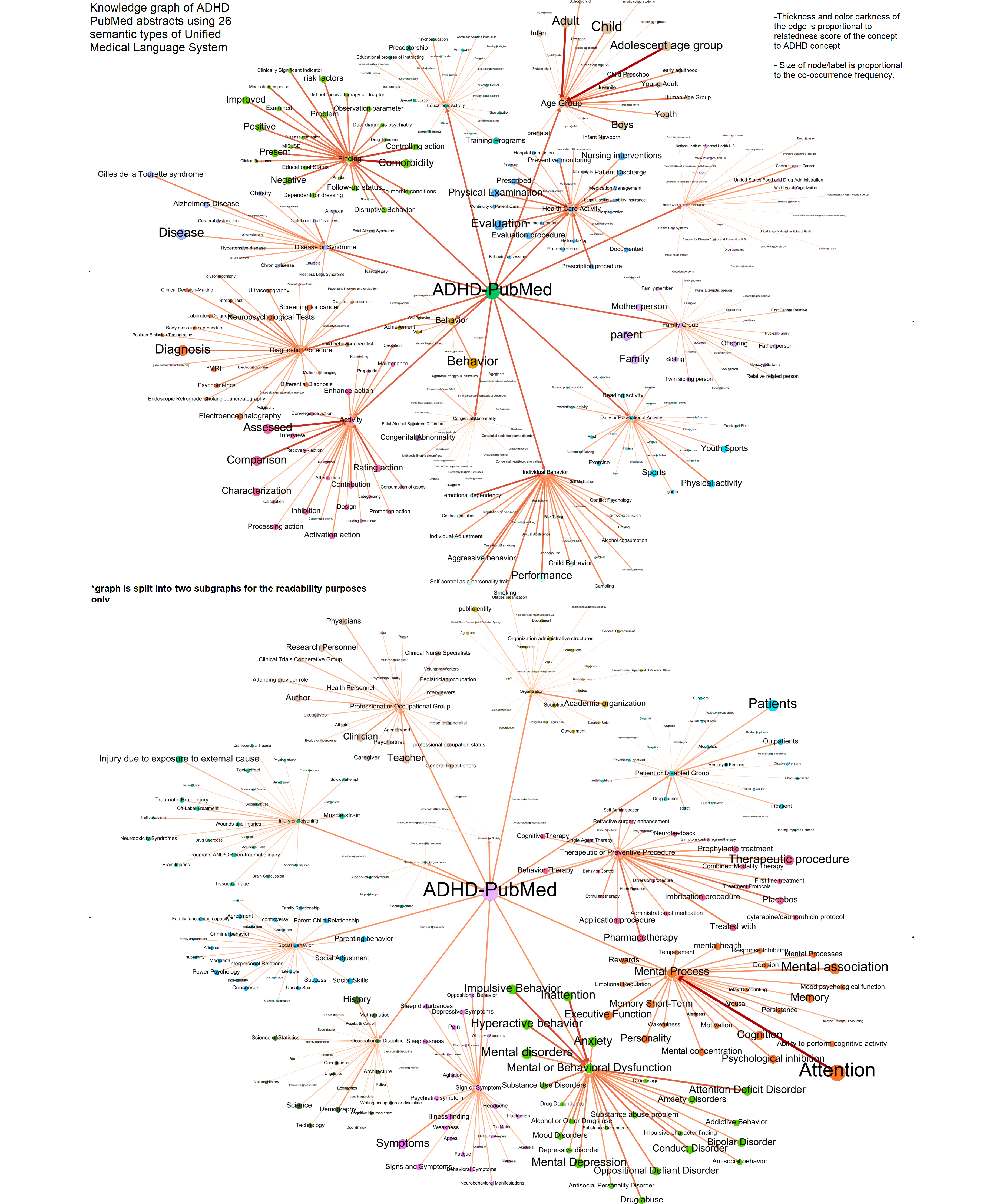

Supplement: Multimedia Appendix 4 [file jmir_v24i8e39888_app4.png]

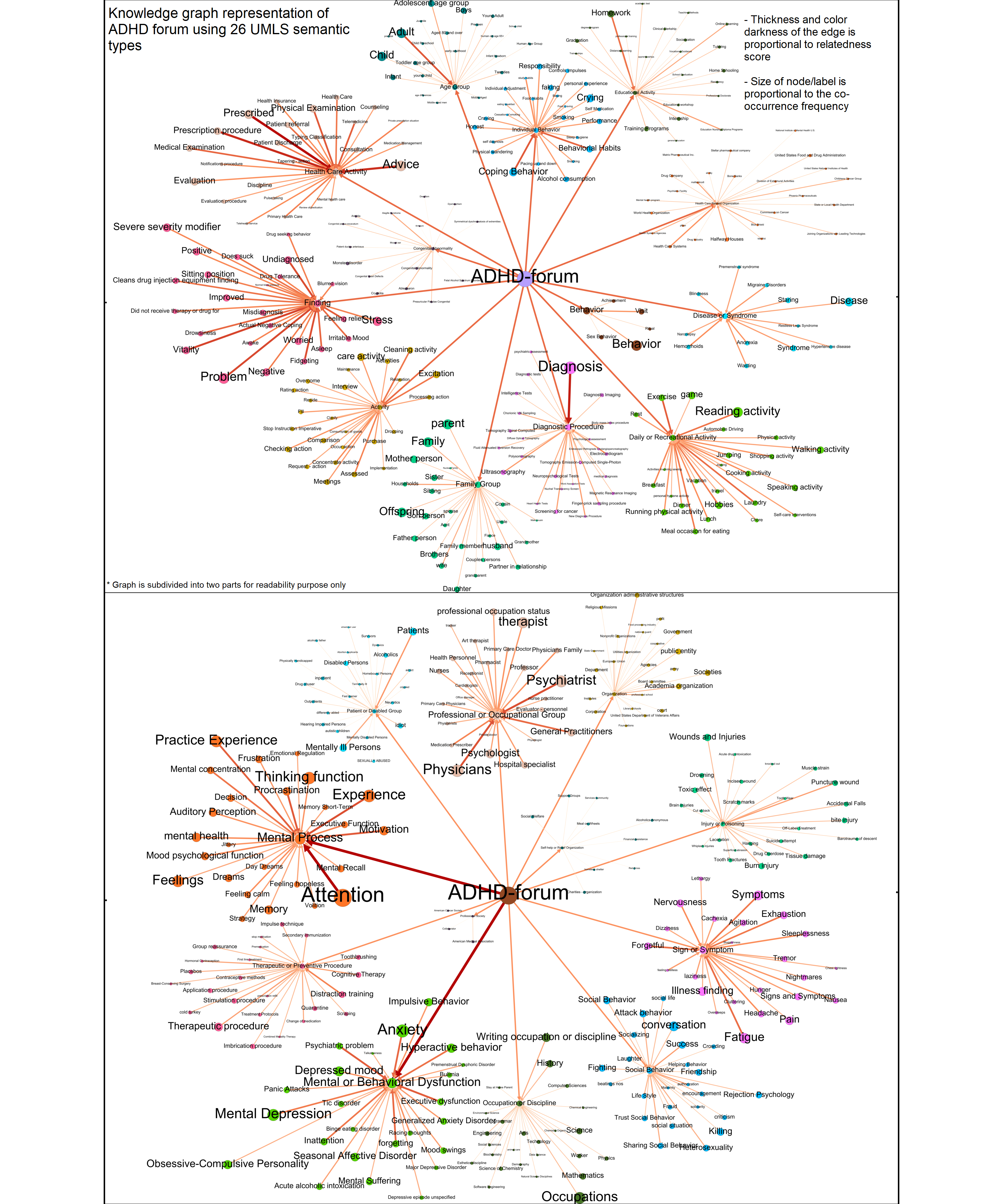

Supplement: Multimedia Appendix 5 [file jmir_v24i8e39888_app5.png]

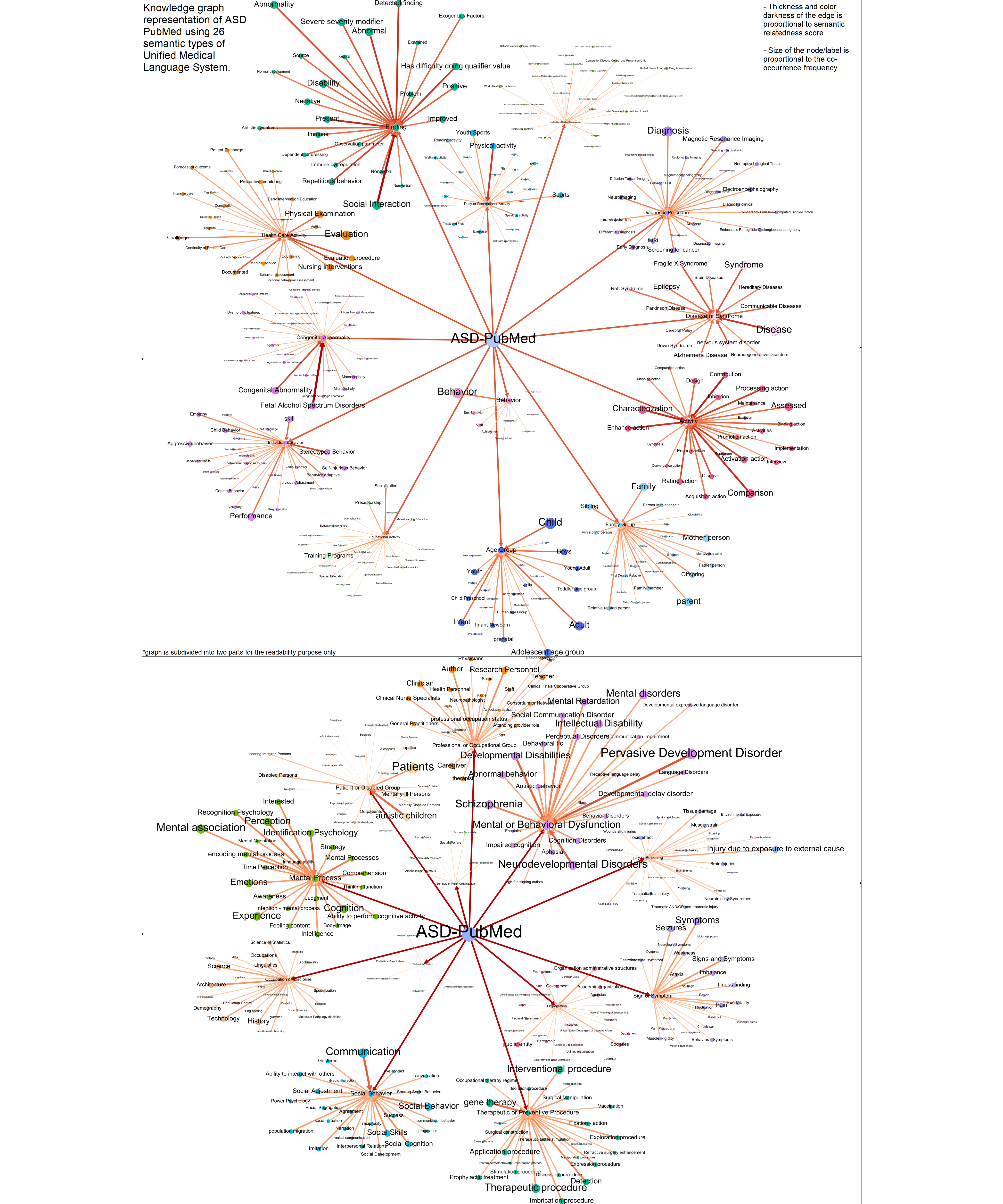

Supplement: Multimedia Appendix 6 [file jmir_v24i8e39888_app6.png]

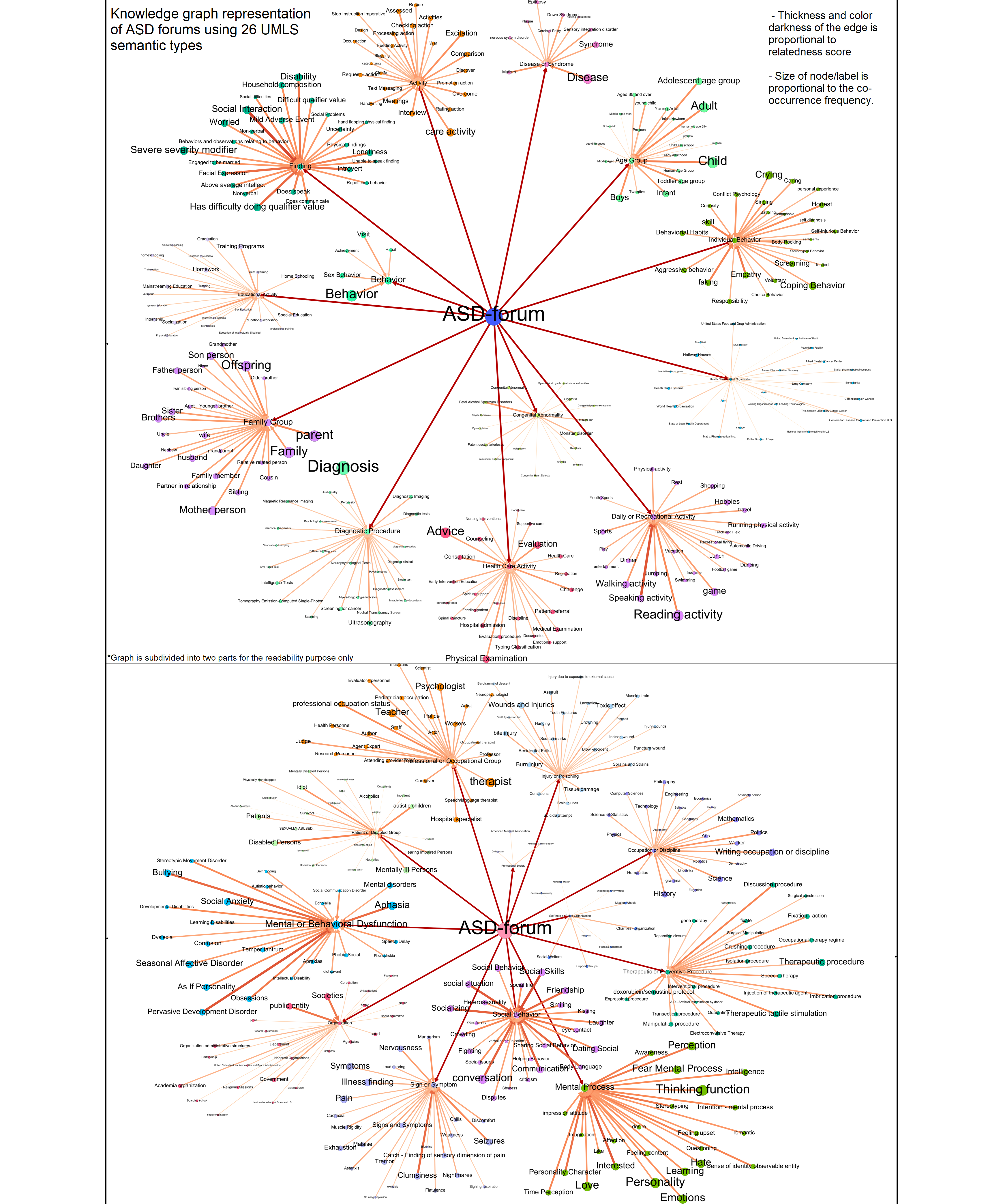

Supplement: Multimedia Appendix 7 [file jmir_v24i8e39888_app7.png]

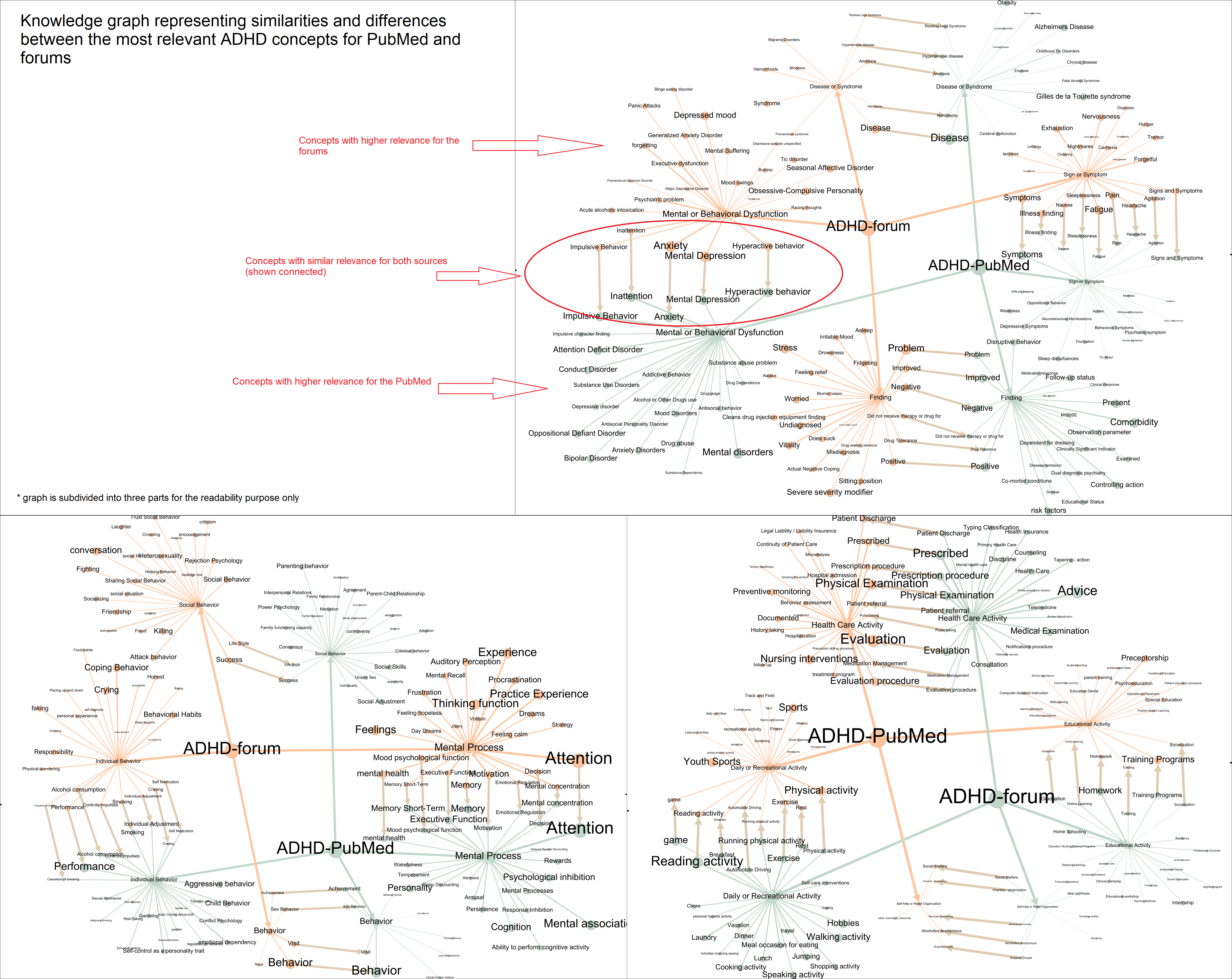

Supplement: Multimedia Appendix 8 [file jmir_v24i8e39888_app8.png]

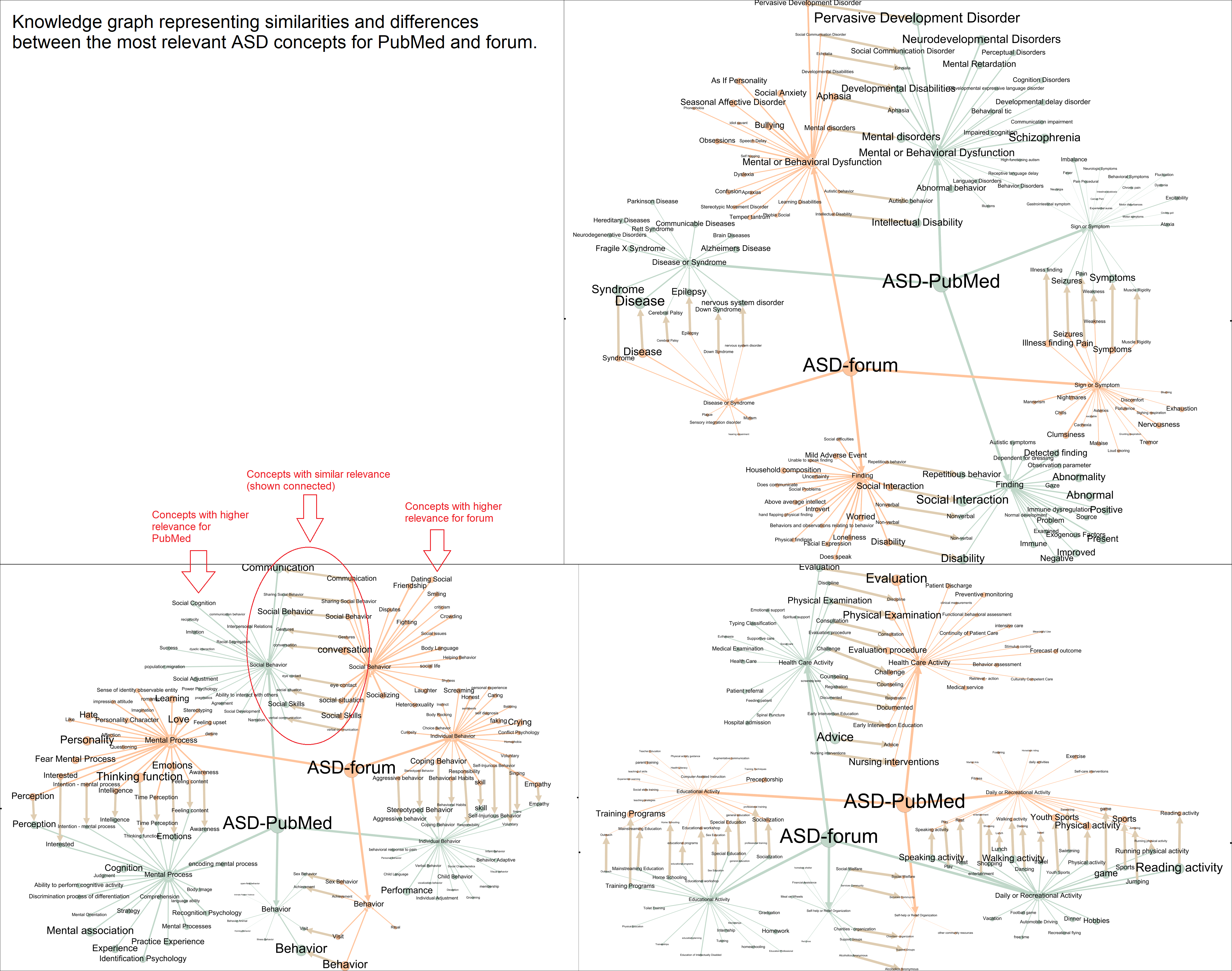

Supplement: Multimedia Appendix 10 [file jmir_v24i8e39888_app10.png]
