# Supplementary material for: Deciphering the Diversity of Mental Models in Neurodevelopmental Disorders: Knowledge Graph Representation of Public Data Using Natural Language Processing
Source: J Med Internet Res. 2022 Aug 5;24(8):e39888. doi: 10.2196/39888 (PMC9391978; doi:10.2196/39888)

Forum - Mapping of UMLS canonical concepts to words in forum text(These concepts are excluded from analysis due to the irrelevant linked words)

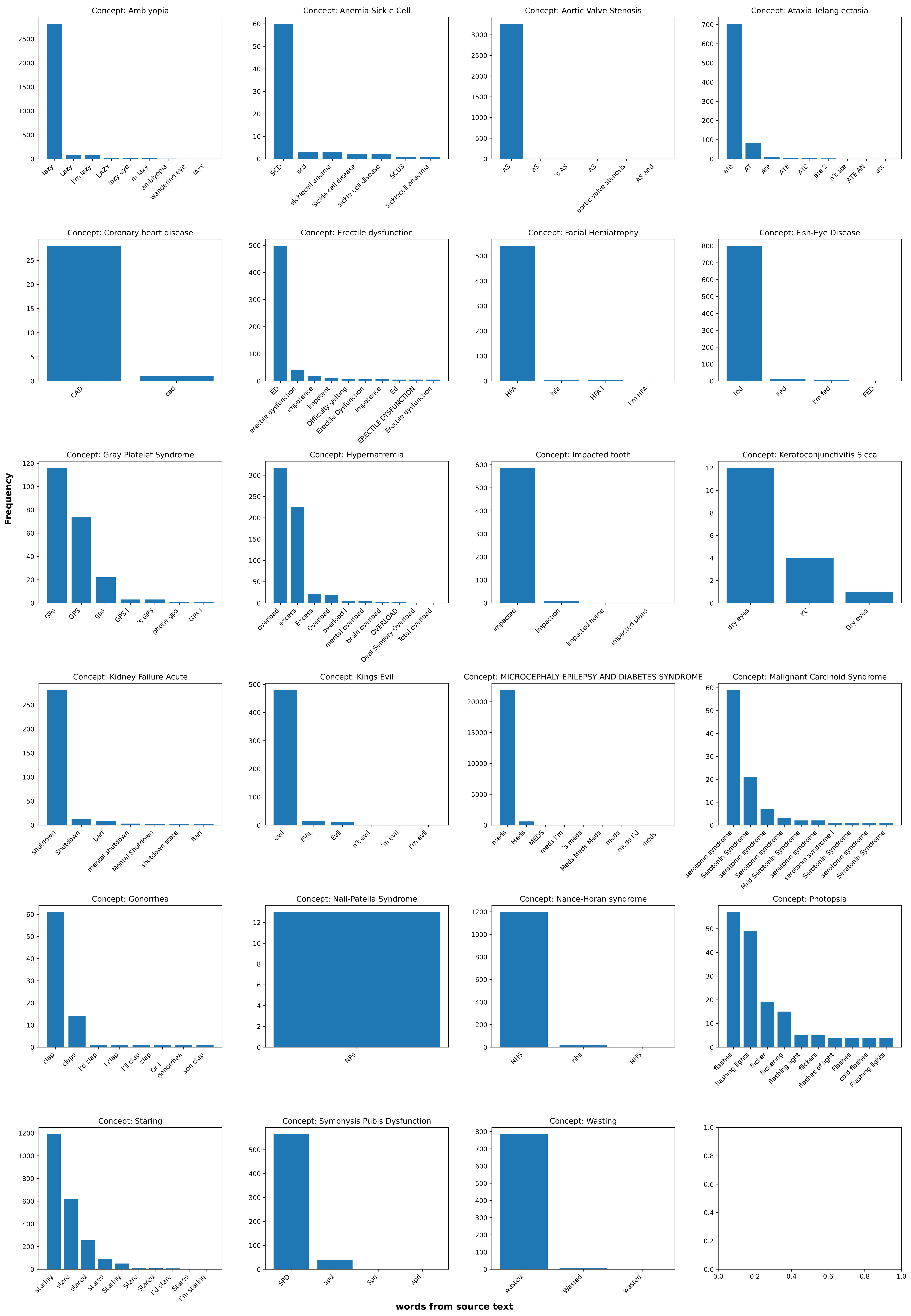

PubMed- Mapping of UMLS canonical concepts to words in PubMed text(These concepts are excluded from analysis due to the irrelevant linked words)

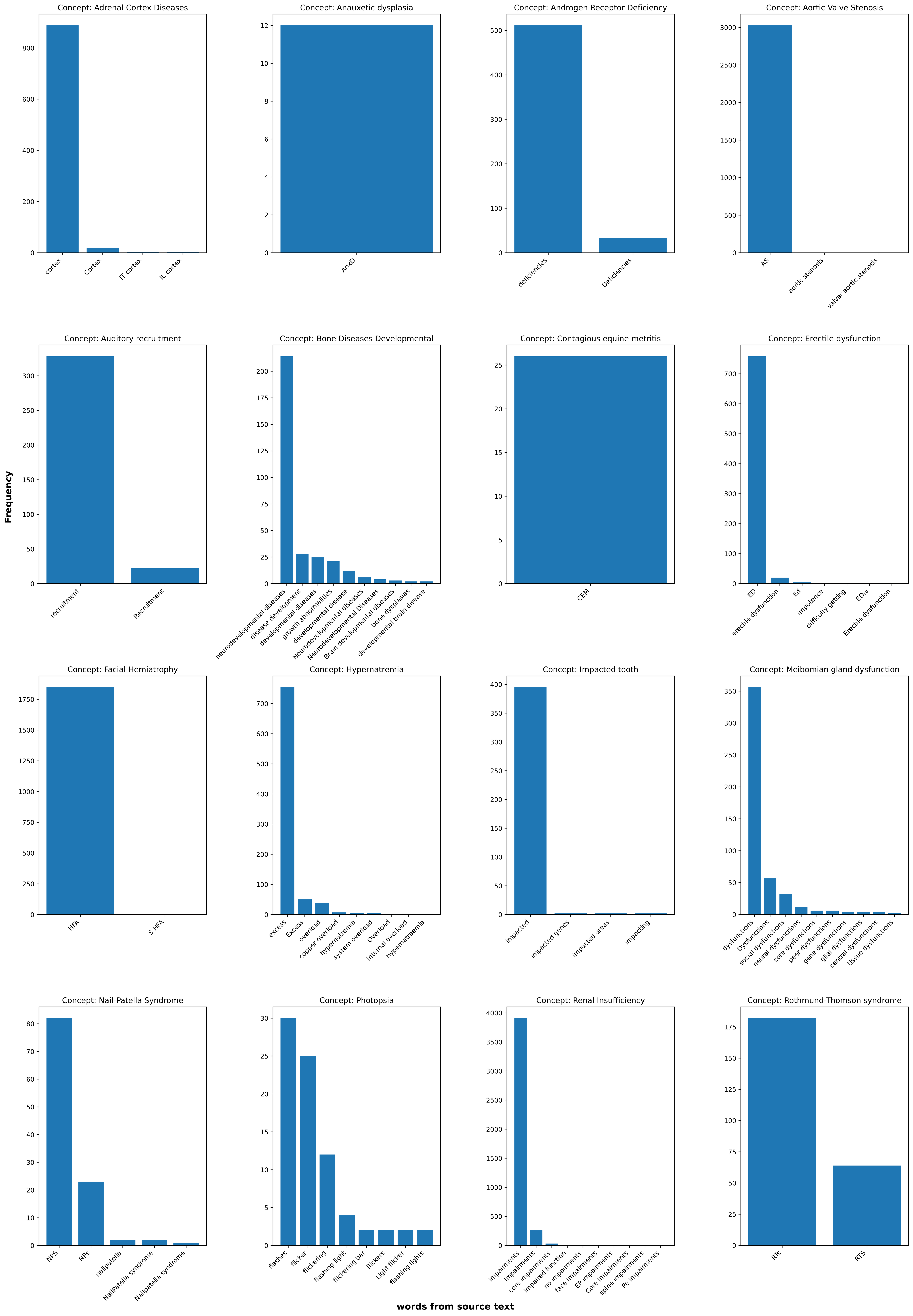

Supplement: Multimedia Appendix 3 [file jmir_v24i8e39888_app3.pdf]
