# Supplementary material for: Deciphering the Diversity of Mental Models in Neurodevelopmental Disorders: Knowledge Graph Representation of Public Data Using Natural Language Processing
Source: J Med Internet Res. 2022 Aug 5;24(8):e39888. doi: 10.2196/39888 (PMC9391978; doi:10.2196/39888)
Supplement: Multimedia Appendix 9 [file jmir_v24i8e39888_app9.pdf]

Comparison of concept relatedness score in forum and PubMed corpus for ADHD

For a given concept:

FR = forum-based relatedness score between the concept and condition (ADHD or ASD)

PR = PubMed-based relatedness score between the concept and condition (ADHD or ASD)

SD = score difference = FR – PR

μ = mean of SD for all the concepts in the corpus

σ = standard deviation of SD

concepts of similar relevance or priority for ADHD forums and PubMed abstracts.

Table S1: Concepts with SD within the range [μ-2*σ, μ+2*σ] representing similar relevance for ADHD forums and PubMed abstracts

**concept**	**FR**	**PR**	**SD**	**SD > μ+2*σ**	**SD < μ+2*σ**
Anxiety	0.65	0.481	0.169	FALSE	FALSE
Sleeplessness	0.522	0.425	0.097	FALSE	FALSE
Mental depression	0.581	0.533	0.048	FALSE	FALSE
Emotional regulation	0.378	0.359	0.019	FALSE	FALSE
Attention	0.999	0.992	0.007	FALSE	FALSE
Training programs	0.217	0.259	-0.042	FALSE	FALSE
Hyperactive behavior	0.614	0.665	-0.051	FALSE	FALSE
Socialization	0.088	0.149	-0.061	FALSE	FALSE
Inattention	0.593	0.771	-0.178	FALSE	FALSE
Impulsive behavior	0.474	0.737	-0.263	FALSE	FALSE

Concepts with higher relevancy/priority for ADHD forums

Table S2: Concepts with SD greater than μ+2*σ representing priorities for ADHD forums

concept	FR	PR	SD	SD > μ+2*σ	SD < μ+2*σ
forgetting	0.643	0.09	0.553	TRUE	FALSE
Exhaustion	0.588	0.05	0.538	TRUE	FALSE
Procrastination	0.697	0.23	0.467	TRUE	FALSE
Psychiatric problem	0.68	0.276	0.404	TRUE	FALSE
study habits	0.391	0	0.391	TRUE	FALSE
Sluggishness	0.387	0	0.387	TRUE	FALSE
Oversleeps	0.367	0	0.367	TRUE	FALSE
Racing thoughts	0.501	0.191	0.31	TRUE	FALSE
Nervousness	0.526	0.24	0.286	TRUE	FALSE
Executive dysfunction	0.687	0.41	0.277	TRUE	FALSE

Concepts with higher relevancy/priority for ADHD PubMed abstracts

Table S3: Concepts with SD less than μ-2*σ representing priorities for ADHD PubMed abstracts

concept	FR	PR	SD	SD > μ+2*σ	SD < μ+2*σ
Anxiety symptoms	0	0.29	-0.29	FALSE	TRUE
regulation of behavior	0	0.308	-0.308	FALSE	TRUE
Amotivation	0	0.324	-0.324	FALSE	TRUE
Sleep phase delay	0	0.327	-0.327	FALSE	TRUE
Addictive behavior	0.145	0.499	-0.354	FALSE	TRUE
Antisocial behavior	0.082	0.454	-0.372	FALSE	TRUE
Substance abuse problem	0.23	0.612	-0.382	FALSE	TRUE
Oppositional behavior	0	0.391	-0.391	FALSE	TRUE
Substance dependence	0	0.443	-0.443	FALSE	TRUE
Conduct disorder	0.033	0.592	-0.559	FALSE	TRUE

Comparison of concept relatedness score in forum and PubMed corpus for ADHD

Concepts of similar relevance or priority for ASD forums and PubMed abstracts

Table S4: Concepts with SD within the range [μ-2*σ, μ+2*σ] representing similar relevance for ASD forums and PubMed abstracts

concept	FR	PR	SD	SD > μ+2*σ	SD < μ+2*σ
Aggressive behavior	0.436	0.269	0.167	FALSE	FALSE
Behavioral tic	0.251	0.445	-0.194	FALSE	FALSE
Developmental disabilities	0.553	0.653	-0.1	FALSE	FALSE
Social communication disorder	0.515	0.569	-0.054	FALSE	FALSE
Mental disorders	0.43	0.418	0.012	FALSE	FALSE
Autistic behavior	0.475	0.458	0.017	FALSE	FALSE
Intellectual disability	0.482	0.458	0.024	FALSE	FALSE
Cerebral palsy	0.317	0.324	-0.007	FALSE	FALSE
Pervasive development disorder	0.73	0.697	0.033	FALSE	FALSE
eye contact	0.606	0.313	0.293	FALSE	FALSE
Aphasia	0.654	0.448	0.206	FALSE	FALSE
Nonverbal	0.513	0.48	0.033	FALSE	FALSE
Repetitious behavior	0.479	0.539	-0.06	FALSE	FALSE
Self-Injurious behavior	0.351	0.26	0.091	FALSE	FALSE
Social Interaction	0.764	0.699	0.065	FALSE	FALSE
Seizures	0.565	0.393	0.172	FALSE	FALSE
Stereotyped behavior	0.384	0.522	-0.138	FALSE	FALSE
Social Skills	0.756	0.489	0.267	FALSE	FALSE
Social behavior	0.456	0.534	-0.078	FALSE	FALSE
Communication	0.697	0.662	0.035	FALSE	FALSE

Concepts with higher relevancy/priority for ASD forums

Table S5: Concepts with SD greater than μ+2*σ representing priorities for ASD forums

concept	FR	PR	SD	SD > μ+2*σ	SD < μ+2*σ
social life	0.536	0.18	0.356	TRUE	FALSE
Crowding	0.566	0.214	0.352	TRUE	FALSE
Introvert	0.617	0.09	0.527	TRUE	FALSE
Mutism	0.488	0.157	0.331	TRUE	FALSE
Phobia social	0.456	0.144	0.312	TRUE	FALSE
Temper tantrum	0.471	0.151	0.32	TRUE	FALSE
Social anxiety	0.611	0.258	0.353	TRUE	FALSE
Nervousness	0.478	0.108	0.37	TRUE	FALSE
Social problems	0.483	0.167	0.316	TRUE	FALSE
Obsessions	0.607	0.14	0.467	TRUE	FALSE
Bullying	0.662	0.129	0.533	TRUE	FALSE

Concepts with higher relevancy/priority for ASD PubMed abstracts

Table S6: Concepts with SD less than range μ-2*σ representing priorities for ASD PubMed abstracts

concept	FR	PR	SD	SD > μ+2*σ	SD < μ+2*σ
Behavior adaptive	0	0.399	-0.399	FALSE	TRUE
Immune dysregulation	0.113	0.441	-0.328	FALSE	TRUE
Social cognition	0	0.502	-0.502	FALSE	TRUE
